# A method for detecting single mRNA molecules in *Arabidopsis thaliana*

**DOI:** 10.1186/s13007-016-0114-x

**Published:** 2016-08-05

**Authors:** Susan Duncan, Tjelvar S. G. Olsson, Matthew Hartley, Caroline Dean, Stefanie Rosa

**Affiliations:** 1 Department of Cell and Developmental Biology, John Innes Centre, Norwich Research Park, Norwich, NR4 7UH UK; 2 Department of Computational and Systems Biology, John Innes Centre, Norwich Research Park, Norwich, NR4 7UH UK

**Keywords:** RNA, FISH, Gene expression, Fluorescence microscopy, Arabidopsis, Transcription

## Abstract

**Background:**

Despite advances in other model organisms, there are currently no techniques to explore cell-to-cell variation and sub-cellular localization of RNA molecules at the single-cell level in plants.

**Results:**

Here we describe a method for imaging individual mRNA molecules in *Arabidopsis thaliana* root cells using multiple singly labeled oligonucleotide probes. We demonstrate detection of both mRNA and nascent transcripts of the housekeeping gene *Protein Phosphatase 2A*. Our image analysis pipeline also enables quantification of mRNAs that reveals the frequency distribution of transcripts per cell underlying the population mean.

**Conclusion:**

This method allows single molecule RNA in situ to be exploited as a powerful tool for studying gene regulation in plants.

**Electronic supplementary material:**

The online version of this article (doi:10.1186/s13007-016-0114-x) contains supplementary material, which is available to authorized users.

## Background

Quantitative real-time PCR is commonly used to analyze plant gene expression, but this method lacks potentially important information relating to sub-cellular localization of RNA and masks cell-to-cell variation [1, 2]. To effectively study these aspects of gene regulation, it is necessary to study RNA at the cellular level.

A method that has achieved this aim is in situ hybridization followed by microscopic analysis. Initially, researchers performed in situ hybridizations using radioactive probes [3]. Early improvements involved linking the probes to enzymes that catalyze chromogenic or fluorogenic reactions [4–6]. In Arabidopsis mRNA in situ hybridization has been routinely used for detailed visualization of gene expression patterns [7–9]. While this method gives good semi-quantitative spatial information, it produces images with limited cellular resolution. More recently plant researchers have used fluorescently labeled probes to directly label transcripts. This has improved cellular resolution, but relatively poor sensitivity has resigned it mainly for detection of highly repetitive RNAs [10, 11]. Single molecule fluorescence in situ hybridization (smFISH) was developed to maximize both sensitivity and specificity by using multiple singly labelled probes to visualize RNA molecules as discrete spots of fluorescence [12]. A recent version of this method uses 48 fluorescently labeled DNA oligonucleotides (20mers) to hybridize to different portions of each transcript. This provides a balance between probe length and number that effectively reduces false positive signals (due to off-target binding) whilst maintaining single molecule sensitivity [13].

Establishment of smFISH in other model systems has led to greater understanding of transcriptional regulation for many genes [14–17]. In addition to quantifying mRNA at the single cell level, this detection method can be used to visualize sites of transcription [18] and long non-coding RNAs [19].

Optical properties of plant cells and tissues provide significant challenges for fluorescence microscopy [20]. Inherent light scattering adversely affects both the excitation and the detection efficiency; moreover plants contain many native molecules that emit high levels of background auto fluorescence compared to other organisms [20]. We chose to develop a smFISH method in fixed Arabidopsis root cells as they typically allow clearer imaging than leaves or other above ground tissue.

We established our method by probing a widely expressed housekeeping gene At1G13320—the A2 scaffolding subunit of *Protein Phosphatase2A* (*PP2A)* [21]. Unlike several environmentally regulated phosphatase subunits, it exhibits mRNA levels that are relatively unperturbed by a range of abiotic and biotic stresses and is transcribed evenly across many tissue types throughout development. These robust properties led to *PP2A* being identified as a superior gene for qPCR normalization [22]. To validate our method we used smFISH to detect *PP2A* mRNAs and used an image analysis pipeline to automate transcript counting within cells. Together our smFISH protocol and image analysis algorithm provides a straightforward framework for other plant researchers to study gene expression at the single-cell and single-molecule resolution.

## Results and discussion

**Fig. 1:**
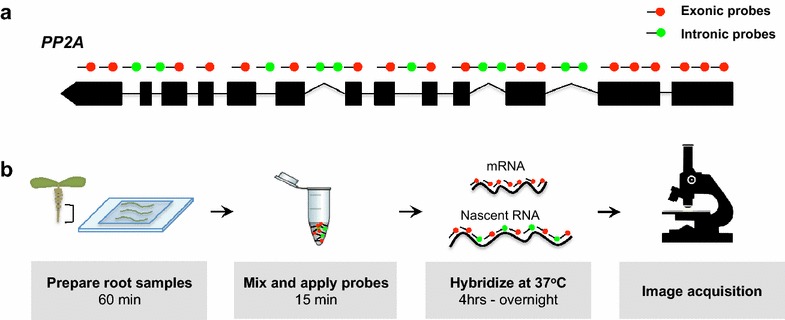
Detecting *PP2A* RNA using single molecule fluorescence In Situ hybridization. **a** Schematic of the probe locations used to detect PP2A RNA. Nascent *PP2A* RNA (*green*) and mRNA (*red*) were detected using probes sets designed to target intronic and exonic RNA sequences respectively. **b** Schematic showing smFISH experimental steps

We designed our initial set of smFISH probes to hybridize exclusively to *PP2A* exons in order to visualize mRNA locations (Fig. 1a). We prepared our samples using a root squash method that typically yields many cells in a single-layer. This method together with the use of red and far-red dyes maximized mRNA signals whilst minimizing background fluorescence.

**Fig. 2:**
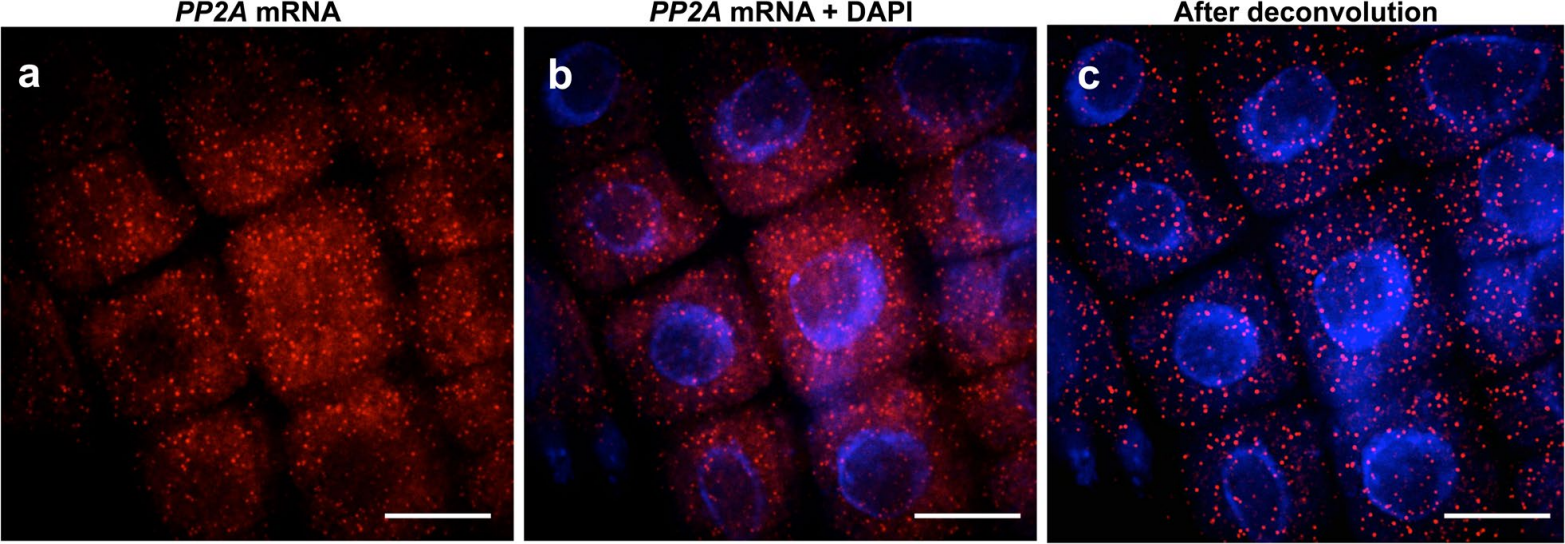
Detection of individual mRNA transcripts in single cells of *Arabidopsis thaliana* roots. Representative maximum projection image of root meristem cell files before (**a**, **b**) and after deconvolution (**c**). *PP2A* mRNA (*red*) and nuclear stain DAPI (*blue*). *Scale bar* = 10 μm

We observed non-specific signals in endo-reduplicated nuclei from the differentiation zone and this restricted our analysis to the meristem region (Additional file 1). Consistent with other reports, we visualized *PP2A* mRNAs as punctate signals 250–300 nm homogeneously dispersed throughout the cytoplasm [13, 23] (Fig. 2a, b, Additional file 2). We found wide-field far superior to confocal microscopy for smFISH imaging with further improvements achieved by deconvolution (Fig. 2c). RNase treatment confirmed that our signals represent RNA locations (Additional file 3).

**Fig. 3:**
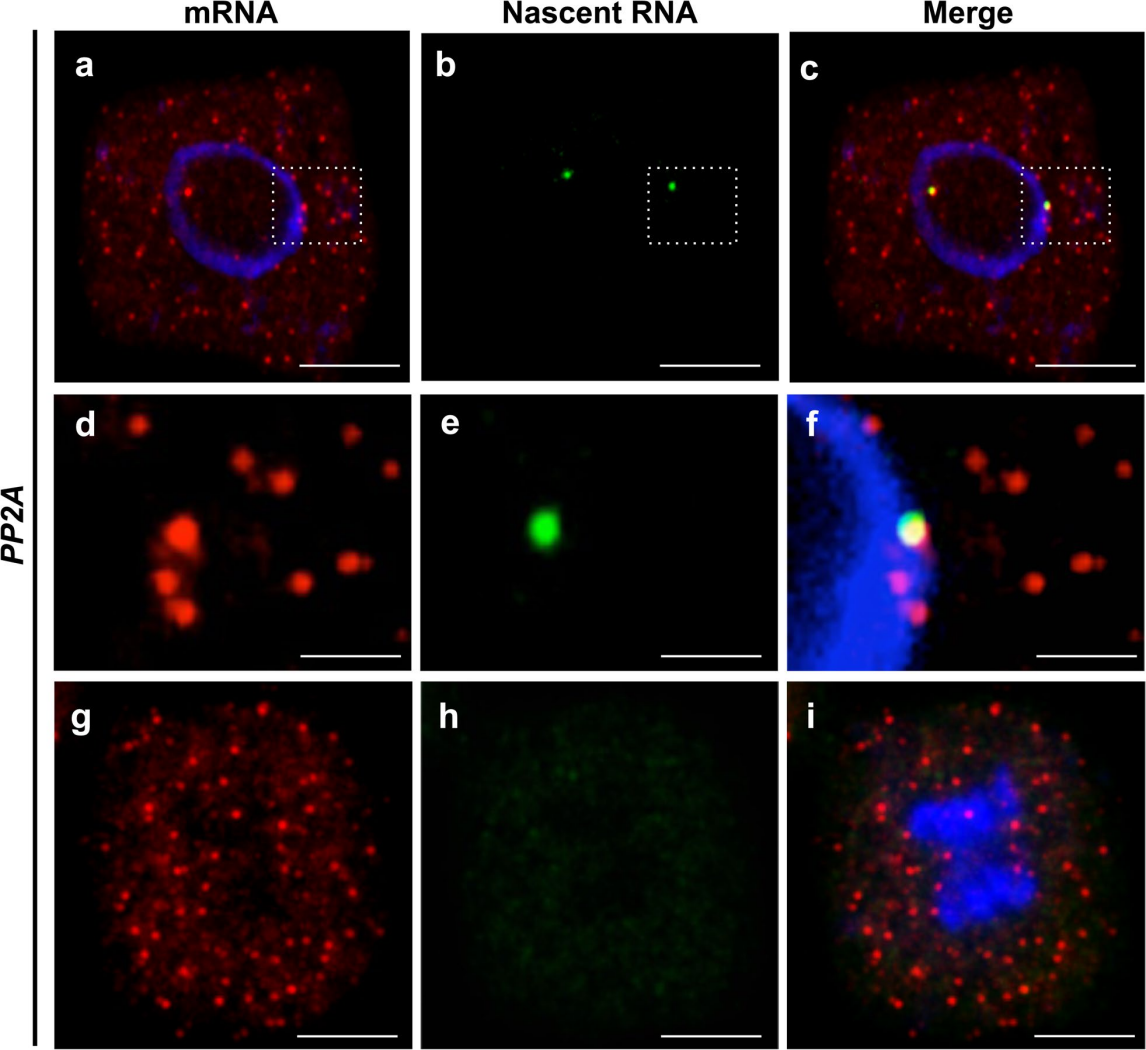
Simultaneous detection of spliced and nascent *PP2A* RNA. Representative images of cells labeled with *PP2A* mRNA (*red*) and nascent *PP2A* RNA (*green*). DNA labeled with DAPI (*blue*). **a**–**c** Representative image of an isolated meristem cell showing cytoplasmic mRNA (**a**); and two sites of active transcription located within the nucleus (**b**). **d**–**f** Magnified image from cell depicted in (**a**–**c**) showing co-localization of nascent *PP2A* RNA and mRNA. **g**–**i** Representative image of a cell during mitosis showing no transcription as judged by the absence of nascent RNA signals (**h**). *Scale Bar* = 10 μm in (**a**–**c**, **g**–**i**) and =0.5 μm in (**d**–**f**)

Next we designed 48 probes to be complimentary only to *PP2A* introns to identify sites of transcription [18] (Fig. 1a). We found that these signals were invariably restricted to the nucleus and co-localized with *PP2A* mRNA foci (Fig. 3a–f; Additional file 4). Also, consistent with RNA production being halted during cell division, we were unable to detect nascent RNA during mitosis (Fig. 3g–i).

We had equal success in imaging RNA labelled with Quasar^®^570 and 670 dyes, but we were unable to observe RNA labelled with FITC (data not shown). We found super-resolution structured illumination microscopy (SIM) produced high quality images of our samples; therefore it may be possible to overcome this multiplex limitation through the detection of spectrally barcoded smFISH probes [24] (Additional file 5).

**Fig. 4:**
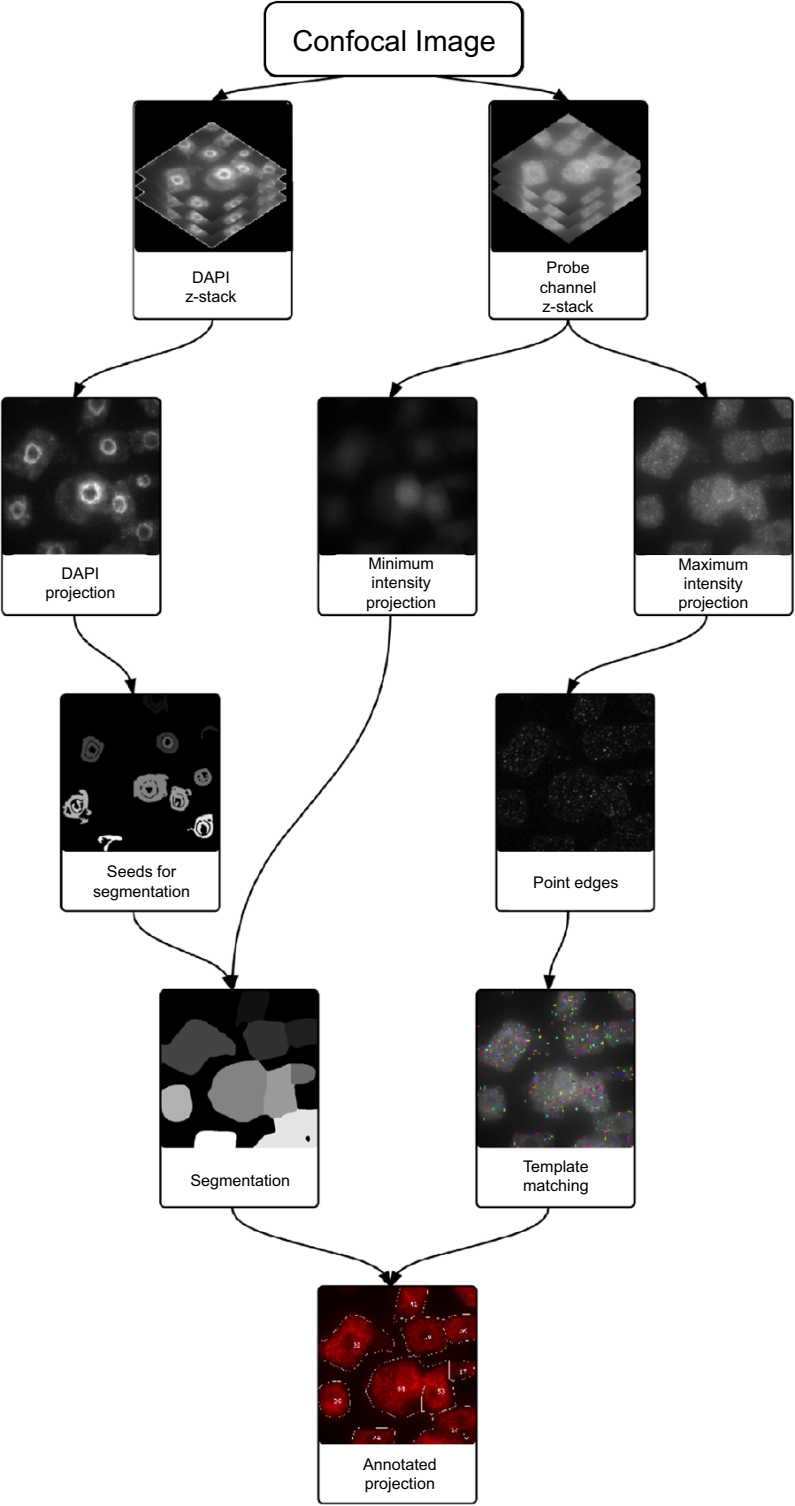
Automated mRNA counting image analysis workflow

**Fig. 5:**
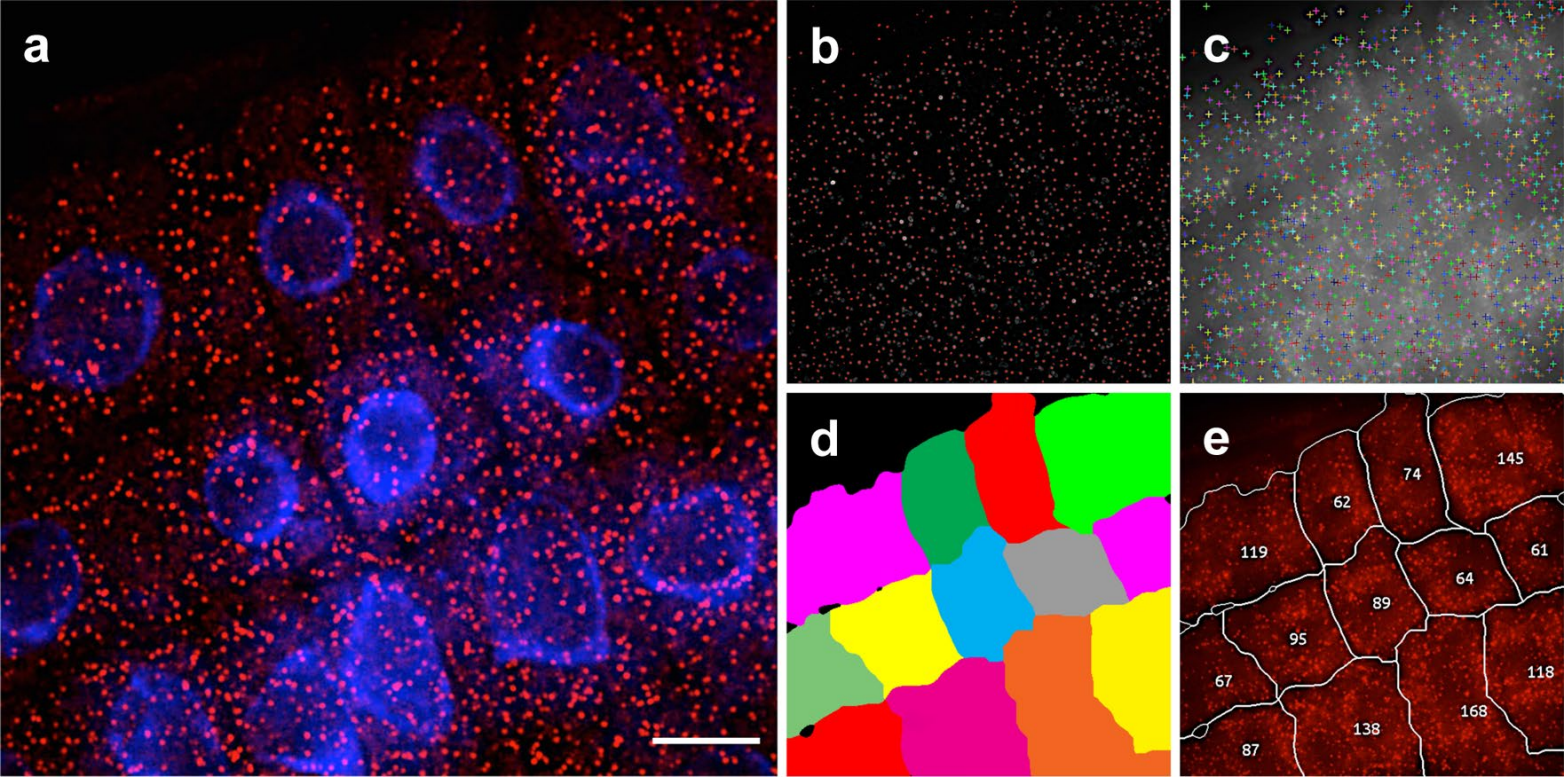
Automated image analysis of *PP2A* mRNA. **a** Representative maximum projection image of cell files labeled with PP2A mRNA probes (*red*). DNA labeled with DAPI (*blue*). **b**, **c** Screen shots showing sequential detection steps used to determine positive mRNA signals. **d** Cell segmentation, where a false-color is rendering individual cells. **e** Output image indicating the number of mRNA signals detected on each cell segmented in (**d**). *Scale bar* = 10 μm

We used an automated image analysis workflow to identify and quantify mRNA in our smFISH images (Fig. 4). Out-of-focus light caused background signal intensity to vary greatly through the image, making it impossible to apply a single uniform threshold level for spot counting. To overcome this we normalized image intensities for each plane of the z-stack before taking a maximum intensity projection. We applied edge detection to this projection and then used template matching to determine the probe locations. This procedure allowed us to avoid having to determine a threshold manually for each image. To obtain cell-level transcript counts we used the watershed algorithm to segment the image into cells using seeds derived from the DAPI nuclear stain channel. We then combined this segmentation with the RNA locations within each segmented cell to generate an annotated image showing derived cell boundaries and transcript counts per cell (Fig. 5).

We chose to perform the analysis on a projection of the z-stack because it simplifies the processing considerably. For data with a higher density of mRNAs or situations where the position of the mRNAs in the z direction is of interest, the spot detection and segmentation algorithms could be implemented in three dimensions. However, for these data, spot density was not high enough to make this necessary.

**Fig. 6:**
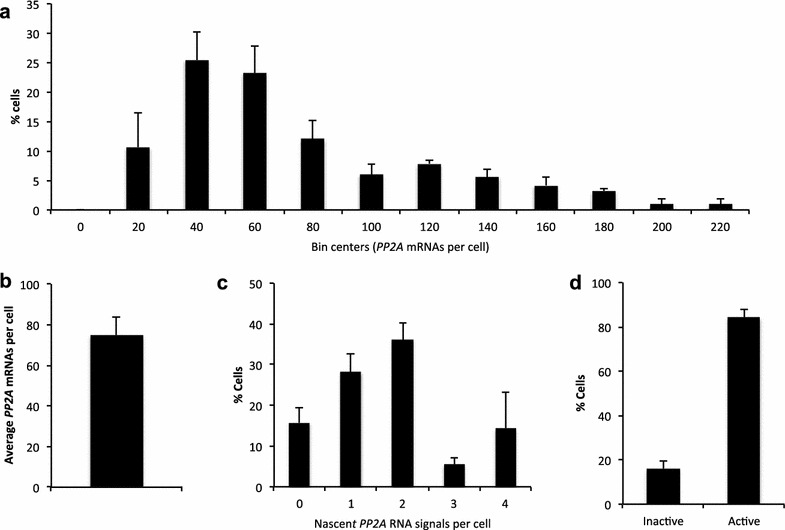
Quantification of mRNA and transcription status for *PP2A*. **a** Frequency distribution of mRNA molecules per cell. **b** Overall average mRNA number per cell. **c** Quantification of active *PP2A* transcription sites as judged by nascent RNA signals per cell. **d** Percentages of transcriptionally active versus inactive cells are shown in **d**. A total of 216 cells were analyzed. *Error bars* = +SEM

Automated analysis of our images revealed that >70 % of cells contain 90 or less *PP2A* mRNA molecules whilst the remaining ~30 % contain between 90 and 220 molecules (Fig. 6a). Every cell we observed contained a minimum of 15 *PP2A* mRNA molecules (Fig. 6a) and an average of 74 mRNAs were detected in each cell (Fig. 6b). Consistent with identification of *PP2A* as a superior normalization gene [22], nascent RNA signals were observed in 84 % of cells. (Fig. 6c, d, Additional file 6).

## Conclusions

In this report we present a FISH method that allows for gene-expression profiling of transcripts in Arabidopsis roots. By characterizing cell-to-cell transcriptional variability of the housekeeping gene *PP2A* we demonstrate that smFISH can be combined with automated image analysis to quantify single RNA transcripts for the first time in plants. As smFISH has been used extensively for RNA analysis in many model organisms [25–28] we believe that this root squash protocol can be easily adapted to suit other plant species with amendments made to the fixation and permeabilization steps as necessary. However adapting smFISH for use in green tissues is likely to represent a greater challenge due to high levels of autofluorescence. Similar issues have been overcome in other organisms through the application of tissue clearing [16] and cryosectioning [29]. We believe that similar approaches may also be employed to enable transcript imaging in other plant tissues.

In addition to quantifying mRNA and visualizing active sites of transcription at the single cell level, corroboratory qRT-PCR data has shown that smFISH can be used to calculate mRNA fold changes at the cellular level [13]. This method can also identify RNA derived from maternal and paternal gene copies [30] and, in conjunction with masking oligonucleotides, it can even distinguish RNA transcripts that differ by only a single nucleotide polymorphism [31]. Our adaptation of smFISH for use in *Arabidopsis thaliana* now opens up these exciting opportunities to the plant research community.

## Methods

### Plant material and growth conditions

Col-0 seeds were surface sterilized in 5 % v/v sodium hypochlorite for 5 min and rinsed three times in sterile distilled water before being sown on MS media minus glucose. They were stratified for 3 days at 5 °C before being transferred to a growth cabinet (Sanyo MLR-351H) 16 h light, 100 μmol m^−2^ s^−1^, 22 °C ± 1 °C.

### Reagents and solutions

Tables 1 and 2 list the oligonucleotide sequences used to detect *PP2A* mRNA and nascent transcripts respectively.

**Table 1:** smFISH probe sequences used to detect PP2A mRNA

*PP2A* exon probes	Sequences (5′–3′)
1	ccgagcgatctatcaatcag
2	gacatcctcaccaaaactca
3	tcgggtataaaggctcatca
4	tagctcgtcgataagcacag
5	ccaagagcacgagcaatgat
6	atcaactcttttcttgtcct
7	catcgtcattgttctcacta
8	atagccaaaagcacctcatc
9	atacagaataaaacccccca
10	caagtttcctcaacagtgga
11	tcatctgagcaccaattcta
12	tagccagaggagtgaaatgc
13	cattcaccagctgaaagtcg
14	ggaaaatcccacatgctgat
15	atattgatcttagctccgtc
16	attggcatgtcatcttgaca
17	aaattagttgctgcagctct
18	gctgattcaattgtagcagc
19	ccgaatcttgatcatcttgc
20	caaccctcaacagccaataa
21	ctccaacaatttcccaagag
22	caaccatataacgcacacgc
23	agtagacgagcatatgcagg
24	gaacttctgcctcattatca
25	cacagggaagaatgtgctgg
26	tgacgtgctgagaagagtct
27	cccattataactgatgccaa
28	tggttcacttggtcaagttt
29	tctacaatggctggcagtaa
30	cgattatagccagacgtact
31	gactggccaacaagggaata
32	catcaaagaagcctacacct
33	ttgcatgcaaagagcaccaa
34	acggattgagtgaaccttgt
35	cttcagattgtttgcagcag
36	ggaccaaactcttcagcaag
37	ggaactatatgctgcattgc
38	gtgggttgttaatcatctct
39	tgcacgaagaatcgtcatcc
40	ttactggagcgagaagcga
41	ctctgtctttagatgcagtt
42	gaacatgtgatctcggatcc
43	catcattttggccacgttaa
44	cgtatcatgttctccacaac
45	atcaacatctgggtcttcac
46	ttggagagcttgatttgcga
47	acacaattcgttgctgtctt
48	cgcccaacgaacaaatcaca

**Table 2:** smFISH probe sequences used to detect *PP2A* nascent transcripts

*PP2A* intron probes	Sequences (5′–3′)
1	actattaccattcttagact
2	gaactgaaactttgtgccgt
3	tgacccattagcctctaaaa
4	ctttaaactcaattccgcct
5	tgcatacatagacaccatca
6	gtaaaccagccttatctaac
7	ttgacagagcatggaaagga
8	tcttctgttttagtggctta
9	acaattgacaaaggacccca
10	gcatatttccaaactttggg
11	acacctataaggggaacact
12	acttcaacctaccaatttcc
13	atgttctcttagatcaacca
14	aaagagcgctaaagccagag
15	tcacatacacaaccacaacc
16	acctataccgaggtatgtat
17	gcttaagtcggtttcacatt
18	acacaatgacagtgttcagt
19	cccataactaggcttgatga
20	acttgcctattacacatcag
21	tgttcaatgcagtaacccta
22	gcttaacttcagctaatggt
23	agctgagatgtagacaaccg
24	ctttcccataaagctcatca
25	agcagctcatacatatctgc
26	aacttcaaccatcactgctt
27	acctctgaagtcagtaatct
28	catggacttccaagtaccaa
29	cacactcttcttaagtgtgt
30	tggtcctttgcataatatga
31	cttagcaaacaccgacagta
32	ctacgtgtagatttataggt
33	atcggtttttaattctgctt
34	gtattcatgatatgagaggc
35	cactccaaactatagagcca
36	atctttatctctaagatgct
37	gatgacagtgactaggacga
38	ccttccaggcacagttaaaa
39	acatagtgaggttttcttat
40	atgccaagttaaaagctgca
41	gagtaacttggtcaatagca
42	acccaatgtcgtacaaagag
43	acagctcctttgaacatgtg
44	tagtcattgacttgaccaaa
45	ggacaaagaatttgctgtca
46	ctggatgattcaatgaaggt
47	ttcaagcagtagagacgaca
48	actccaataaccaatagcta

#### Liquid nitrogen

Nuclease-free water—not DEPC treated (Qiagen, Cat. No. 129117).

Paraformaldehyde (Sigma, Cat. No. P6148) freshly depolymerized, 4 % w/v in water.

Nuclease-free 10× Phosphate Buffered Saline (Thermo Scientific, Cat. No. AM9624).

70 % Ethanol (freshly made using nuclease free water).

Nuclease-free 20× saline-sodium citrate (20× SSC, Thermo Scientific, Cat. No. AM9763).

RNase A (Sigma, Cat. No. R4642) diluted to 100 μg/ml.

T_10_E_1_ buffer (10mM Tris-HCl, 1mM EDTA, pH 8)—Sigma, Cat. No. 93283-100mL  

Deionized Formamide (Sigma, Cat. No. F9037).

Dextran Sulphate (Sigma, Cat. No. Res2029D).

Nuclease free Tris HCl buffer 1 M pH8 (Thermo Scientific, Cat. No. AM9855G).

Glucose oxidase (Sigma,Cat. No. G0543).

Bovine Live Catalase (Sigma, Cat. No. C3155).

### Wash buffer (50 ml)

5 ml nuclease free 20× SSC mixed with 5 ml nuclease free deionized formamide and nuclease free water up to 50 ml final volume. (Final composition: 10 % formamide, 2× SSC).

**DAPI** (4′, 6-Diamidino-2-phenylindole; Sigma cat. no. D9564) Diluted to 100 ng/μl in wash buffer (Final composition: 100 ng/μl, 10 % formamide, 2× SSC).

### Hybridization solution (10 ml)

Dissolve 1 g dextran sulfate in 1 ml nuclease free 20× SCC, 1 ml deionized formamide and nuclease free water up to 10 ml final volume. (Final composition: 100 mg/ml dextran sulfate and 10 % formamide in 2× SSC).

### Anti-fade GLOX buffer minus enzymes (1 ml)

40 μl 10 % glucose in nuclease-free water, 10 μl 1 M Tris–HCl, pH 8.0 and 100 μl 20× SCC was mixed with 850 μl nuclease-free water. (Final composition: 0.4 % glucose in 10 nM Tris–HCl, 2× SSC).

### Anti-fade GLOX buffer containing enzymes (100 μl)

1 μl glucose oxidase and 1 μl mildy vortexed catalase suspension added to 100 μl GLOX minus enzyme solution.

### Equipment

#### Razor blades

##### Forceps

Poly-l-Lysine slides (Sigma, Cat. No. PO425 or similar NOTE: these are not essential but the samples adhere better to these than untreated slides).

Low stender-form preparation dishes (VWR, Cat. No. 470144-866 or similar).

22 mm × 22 mm No.1 glass coverslips (Fisher Scientific, Cat. No. 12333128 or similar).

Coplin jar (Sigma, Cat. No. S6016 or similar).

Parafilm^®^ M sealing film (Bemin, Cat. No. PM992).

##### Orbital shaker

Hybridization chamber (or a suitable dark box with a layer of tissue moistened with water will suffice).

##### 37 °C incubator

Zeiss Elyra PS1 inverted microscope with cooled EM-CCD Andor iXon 897 camera.

### smFISH probe design

Since designing smFISH probes is similar to designing PCR primers most primer design software packages can be used [23] but we used the online program Stellaris^®^ Probe Designer version 2.0 from Biosearch Technologies (http://singlemoleculefish.com). Input of *PP2A* coding sequence into the program automatically generates a set of probes complementary to the *PP2A* mRNA, optimized for binding to the target sequence. Before ordering our pre-labelled probes from Biosearch Technologies we completed a TAIR BLAST query for each sequence to ensure target specificity (https://www.arabidopsis.org/Blast/). Tables 1 and 2 list the oligonucleotide sequences used to detect *PP2A* mRNA and nascent transcripts respectively.

### Sample preparation (timing: 2 h)

Seedlings were removed from the media 10 days after germination. Root tips were dissected using a razor blade and forceps and placed into a glass dish containing 4 % paraformaldehyde to fix for 30 min at room temperature. The roots were removed from the fixative and washed twice with 1× PBS. 3–4 roots were then arranged on a slide and covered by a glass coverslip and the meristems were squashed manually by applying pressure through the coverslip. The slide, together with the sample and coverslip, were then submerged briefly in liquid nitrogen (~5 s) to adhere the roots to the slide. The coverslip was then flipped off with a razor blade and the samples were left to dry at room temperature for a minimum of 30 min. Tissue permeabilization was then carried out by immersing the samples in coplin jars containing 70 % ethanol and left to shake gently for a minimum of 1 h.

Note: We ensured coplin jar lids were sealed with parafilm to prevent evaporation during the ethanol incubation period.

### Hybridization (timing: 4 h—overnight)

Residual ethanol was left to evaporate at room temperature for 5 min before 2, 2-min washes were carried out with wash buffer. 100 μl of hybridization solution with probes at a final concentration of 250 nM was then added to each slide. Coverslips were laid over the samples to prevent buffer evaporation and the probes were left to hybridize in a humid chamber at 37 °C overnight in the dark.

### Sample mounting (timing: 2 h)

Hybridization solution containing unbound probes was removed using a pipette in the morning. Each sample was then washed twice with 200 μl wash buffer and finally immersed in coplin jars containing wash buffer for 30 min at 37 °C. 100 μl of the nuclear stain DAPI was then added to each slide and left to incubate at 37 °C for 30 min. Following DAPI removal, 100 μl 2× SSC was added samples and removed. 100 μl GLOX buffer minus enzymes was added to the samples and left to equilibrate for 2 min and then replaced with 100 μl of anti-fade GLOX buffer containing enzymes. The samples were then covered by coverslips sealed. Excess GLOX buffer was wicked away using tissue before the coverslips were sealed with nail varnish. We immediately imaged our samples as we observed a noticeable reduction in image quality around 4 h after mounting.

Note: Oxygen-scavenging GLOX buffer maximised the stability of our smFISH fluorophores and we observed rapid bleaching when it was substituted with the commercial anti-fade mounting media Vectorshield (data not shown).

### Image acquisition

A Zeiss Elyra PS1 inverted microscope was used for imaging. A 100X oil-immersion objective (1.46 NA) and cooled EM-CCD Andor iXon 897 camera (512 × 512 QE > 90 %) was used to obtain all images in the standard, rather than super-resolution mode. The following wavelengths were used for fluorescence detection: for probes labeled with Quasar^®^570 an excitation line of 561 nm was used and signal was detected at 570–640 nm; for probes labeled with Quasar^®^670 an excitation line of 642 nm and signal was detected at 655–710 nm; for DAPI an excitation line of 405 nm and signal was detected at wavelengths of 420–480 nm. For all experiments exposure times between 200–250 ms were used and a series of optical sections with z-steps of 0.2 μm were collected.

Note: When establishing this technique for the first time we recommend that the following controls be carried out: no probe (where probes are omitted from the hybridization solution) Additional file 1, and RNase A treatment (Additional file 2). To confirm RNA specificity we incubated samples with RNase for 1 h at 37 **°**C in a humid chamber after the ethanol permeabilization step, rinsed in 10 mM HCl for 5 min, washed twice with 2× SSC for 5 min before the protocol was continued.

Z-stacks were deconvolved using AutoQuant X2 (Media Cybernetics). Projections and analysis of 3D pictures were performed using Fiji (an implementation of ImageJ, a public domain program by W. Rasband available from http://rsb.info.nih.gov/ij/). Typically from 4 to 6 roots more than 300 cells can be obtained by this method, which were then suitable for further analysis using our automated mRNA counting programme.

### Structured illumination microscopy

A Zeiss Elyra PS1 inverted microscope was used for imaging using a 63X water objective (1.2 NA) to match samples mounted in GLOX buffer. The SIM camera used was an EM-CCD Andor iXon 885. We collected ×5 phases at ×3 angles total 15 images per plane. Series of optical sections with z-steps of 0.2 μm were collected.

The following wavelengths were used for fluorescence detection: for probes labeled with Quasar^®^570 an excitation line of 561 nm was used and signal was detected at 570–640 nm; for probes labeled with Quasar^®^670 an excitation line of 642 nm and signal was detected at 655–710 nm; for DAPI an excitation line of 405 nm and signal was detected at wavelengths of 420–480 nm. For all experiments series of optical sections with z-steps of 0.2 μm were collected.

Images were processed using Zen Black default parameters. The images were also colour aligned using Zeiss “channel aligned” tool. Reference images of multiple coloured beads were collected in SIM mode then processed. Then an alignment matrix was generated using the SIM bead data and this was applied to the experimental SIM data.

## Image analysis

We have made our mRNA counting programme publically available at: https://github.com/JIC-CSB/FISHcount. Our smFISH image analysis consists of two components—cell segmentation and mRNA counting. These combine into an overall workflow that results in an image where each cell is annotated with the number of mRNA located within it (Figs. 4, 5). Bioformats [32] is used to convert the microscope image into individual z-stacks for each channel. The analysis pipeline then processes these z-stacks to produce the annotated image, and is implemented in the Python programming language [33].

### Segmentation

The Watershed algorithm is used to segment the image into regions representing cells, using the implementation provided by the scikit-image library [34]. Segmentation using the Watershed algorithm requires an input image denoting gradient magnitude, and a set of seeds for initialising the flood filling of the input image.

To identify the seeds for the Watershed algorithm each plane in the DAPI stack is normalised for intensity, then a maximum intensity projection taken. Contrast Limited Adaptive Histogram Equalization (CLAHE, [35]) is used to locally equalize the intensity of the projection. A Sobel filter is applied to the projection to find nuclear edges. Otsu’s thresholding is then applied to select the nuclei. Each detected nucleus is reduced to its centroid for use as a seed for the segmentation.

The gradient magnitude input for the Watershed algorithm is generated by taking a minimum intensity projection of the probe channel, which represents the background auto-fluorescence of each cell. This projection is equalized with CLAHE and smoothed with a Gaussian filter. Taking this image as the basis for the Watershed algorithm and applying the seeds derived from the DAPI channel yields a segmented image.

### mRNA counting

To locate the spots representing RNA molecules, each z-slice in the probe channel stack is normalised, and a maximum intensity projection of the stack taken. A Sobel filter is applied to the projection to detect edges. We use scikit-image’s implementation of fast normalised cross-correlation template matching to find the probe locations. This algorithm tests the correlation between a given template and the equivalently sized section of a larger image for each point in that image. It produces another image, the intensity values of which correspond to the degree to which the template correlates with the image (so that the maximum intensity value corresponds with perfect correlation, and the minimum with perfect anti-correlation). We initially apply this algorithm using a template constructed as an annular element sized to the diffraction radius of the microscope. The single closest match to this template is then taken as a second template to re-apply the correlation. We then apply a correlation threshold, correlation values above which corresponded to identified mRNA spots, yielding their locations. This threshold was chosen based on comparison to manual spot counting in test data sets, such that it gave an optimum balance between false negatives and false positives.

For validation of the results, identified spot locations and the segmentation derived from the DAPI nuclear stain and probe autofluorescence is used to produce an annotated image. This image overlays probe counts and segmentation boundaries on the projection of the probe autofluorescence channel. Each image is manually inspected to ensure that the image analysis workflow has not generated spurious results.

Graphs presented in Fig. 6 were created using GraphPad Prism 6 for Mac OS X software version 6.0 g (La Jolla, California).

## Additional files

**Additional File 1.** Arabidopsis root meristem cells are suitable for smFISH analysis. Representative images of nuclei from root meristem (a-c) and differentiation zone (d-f) in the absence of probe labeling. Non-specific signals were observed in endoreduplicated cells from the differentiation zone, in both red (d) and far-red channels (e). DNA labeled with DAPI (blue). Scale bar = 8 μm.

**Additional File 2.** Spot measurements. (a) Images of *PP2A* RNA spots visualized using Quasar570^®^ and Quasar670^®^ filter channels. (b) Line scans of fluorescent intensity corresponding to the lines shown in (a). Each line scan corresponds to the different fluorophores. The red linescan corresponds to analysis performed for a *PP2A* mRNA spot labeled with Quasar570^®^ and the green linescan to *PP2A* unsliced RNA labeled with Quasar670^®^ probes.

**Additional File 3.** mRNA signals are undetectable following RNase treatment. Representative images of RNase treated cells labeled with *PP2A* mRNA probes (red). DNA labeled with DAPI (blue). Scale bar = 8 μm.

**Additional File 4.** Additional examples of simultaneous detection of spliced and nascent *PP2A* RNA. Nuclei are labeled with the nuclear stain DAPI (blue), *PP2A* mRNA (red) and nascent *PP2A* RNA (green). Scale bars = 10 μm.

**Additional File 5.** *PP2A* mRNA imaged using Structured Illumination Microscopy. Nuclei are labeled with the nuclear stain DAPI (blue), *PP2A* mRNA (red) and nascent *PP2A* RNA (green). Scale bar = 10 μm.

**Additional File 6.** Raw data for PP2A mRNA and nascent counts.
