# Supplementary figures and images for: A method for detecting single mRNA molecules in Arabidopsis thaliana
Source: Plant Methods. 2016 Aug 5;12:13. doi: 10.1186/s13007-016-0114-x (PMC5192599; doi:10.1186/s13007-016-0114-x)

570nm

670nm

DAPI

**a**

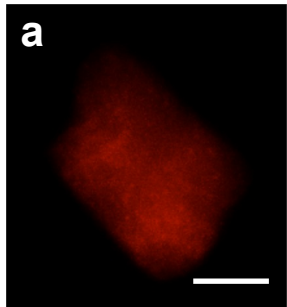

**b**

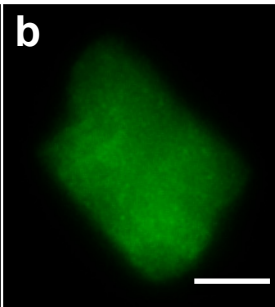

**c**

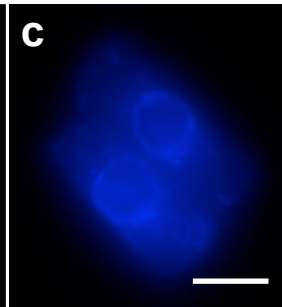

**d**

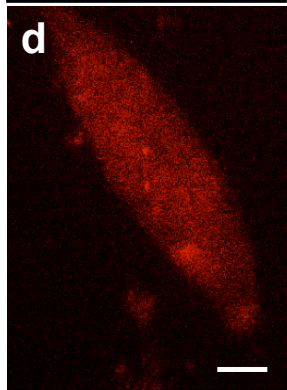

**e**

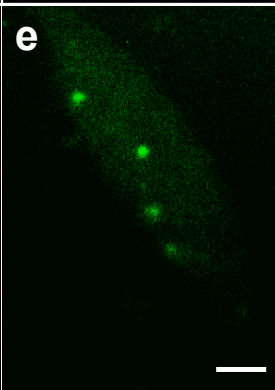

**f**

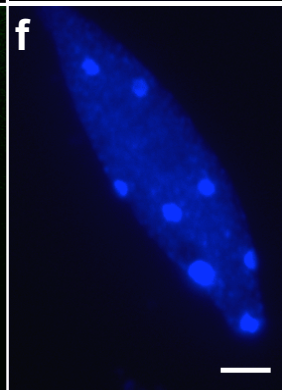

No probe control

Supplement: Supplementary file 1 — Additional File 1. Arabidopsis root meristem cells are suitable for smFISH analysis. Representative images of nuclei from root meristem (a-c) and differentiation zone (d-f) in the absence of probe labeling. Non-specific signals were observed in endoreduplicated cells from the differentiation zone, in both red (d) and far-red channels (e). DNA labeled with DAPI (blue). Scale bar = 8 μm. [file 13007_2016_114_MOESM1_ESM.pdf]

**a****Merge**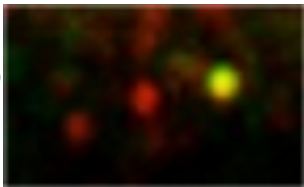**Quasar 570**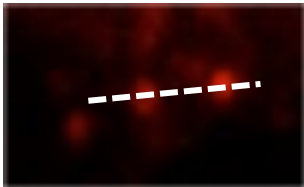**Quasar 670**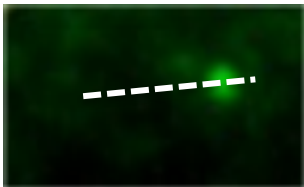**b**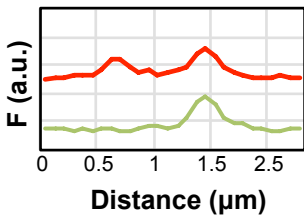

Supplement: Supplementary file 2 — Additional File 2. Spot measurements. (a) Images of PP2A RNA spots visualized using Quasar570® and Quasar670® filter channels. (b) Line scans of fluorescent intensity corresponding to the lines shown in (a). Each line scan corresponds to the different fluorophores. The red linescan corresponds to analysis performed for a PP2A mRNA spot labeled with Quasar570® and the green linescan to PP2A unsliced RNA labeled with Quasar670® probes. [file 13007_2016_114_MOESM2_ESM.pdf]

**RNase treated**

***PP2A* mRNA**

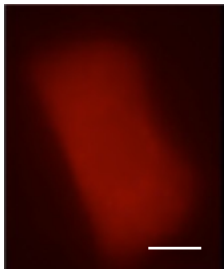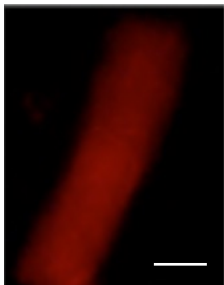

***PP2A* mRNA  
and DAPI**

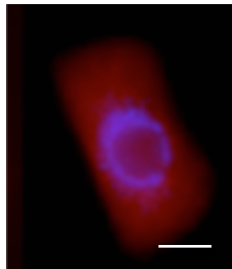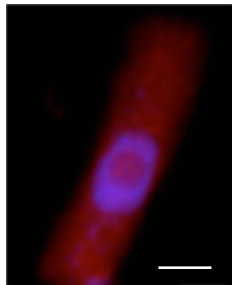

Supplement: Supplementary file 3 — Additional File 3. mRNA signals are undetectable following RNase treatment. Representative images of RNase treated cells labeled with PP2A mRNA probes (red). DNA labeled with DAPI (blue). Scale bar = 8 μm. [file 13007_2016_114_MOESM3_ESM.pdf]

mRNA

Nascent RNA

Merge

PP2A

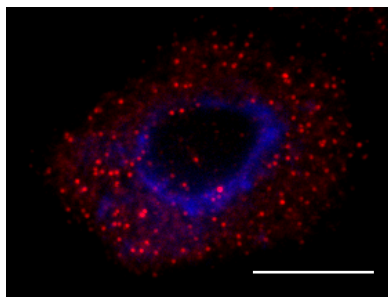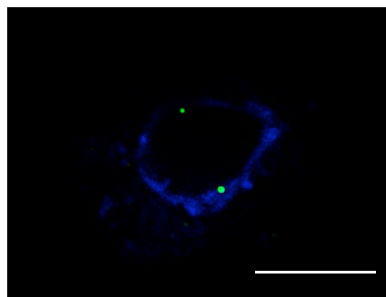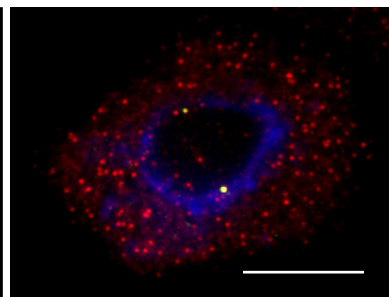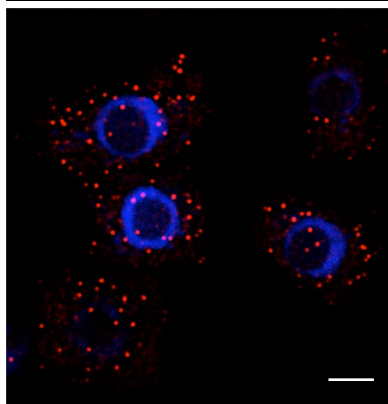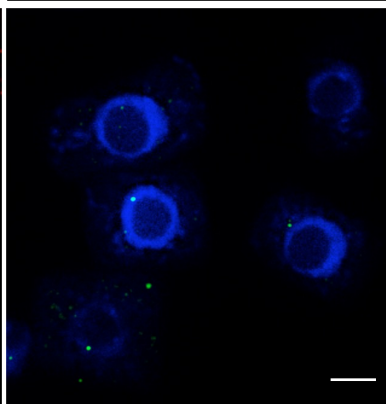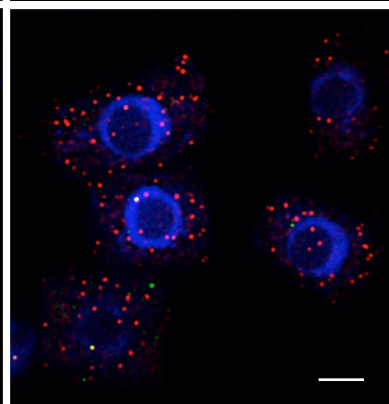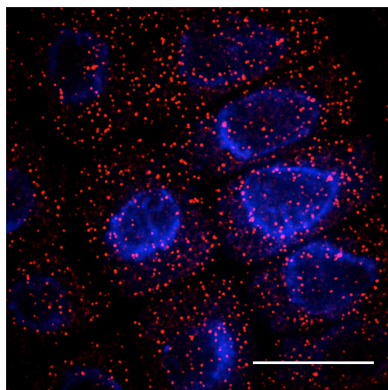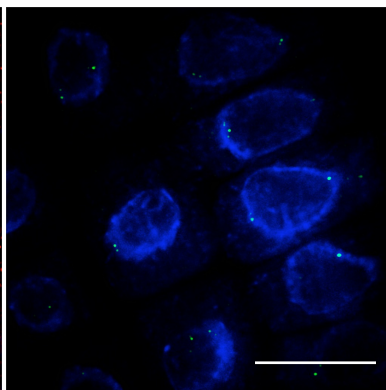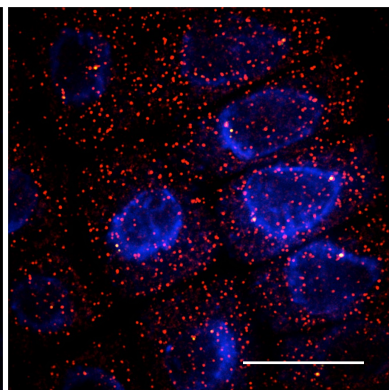

Supplement: Supplementary file 4 — Additional File 4. Additional examples of simultaneous detection of spliced and nascent PP2A RNA. Nuclei are labeled with the nuclear stain DAPI (blue), PP2A mRNA (red) and nascent PP2A RNA (green). Scale bars = 10 μm. [file 13007_2016_114_MOESM4_ESM.pdf]

*PP2A* mRNA

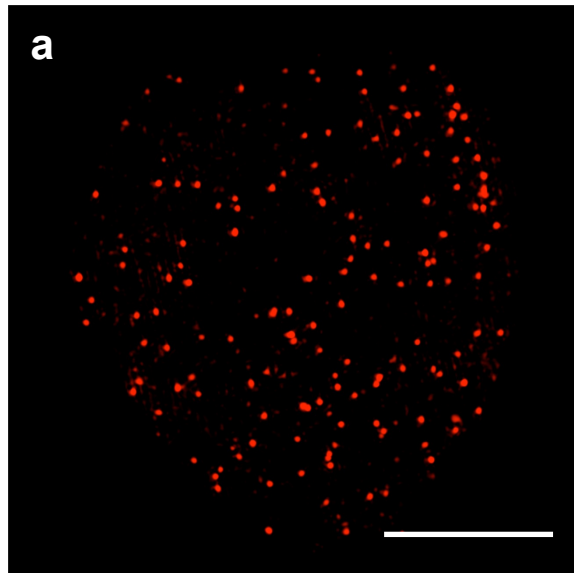

Nascent *PP2A* RNA

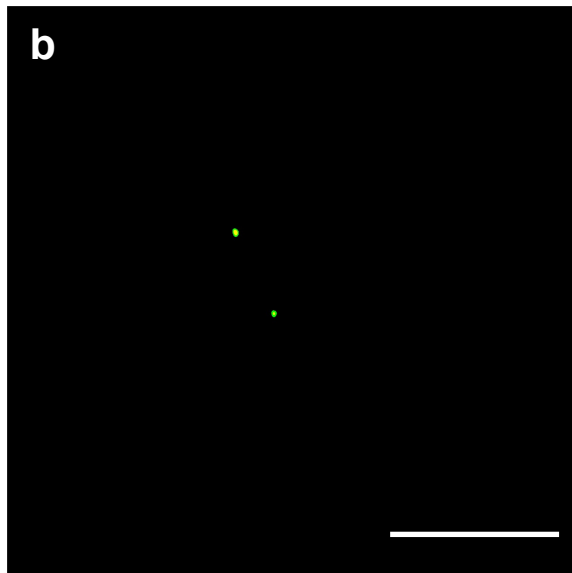

Merge with DAPI

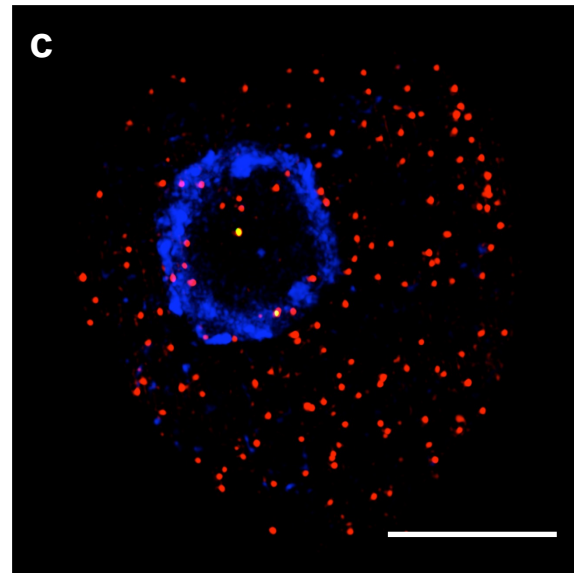

Supplement: Supplementary file 5 — Additional File 5. PP2A mRNA imaged using Structured Illumination Microscopy. Nuclei are labeled with the nuclear stain DAPI (blue), PP2A mRNA (red) and nascent PP2A RNA (green). Scale bar = 10 μm. [file 13007_2016_114_MOESM5_ESM.pdf]
